# Bioconversion of biphenyl to a polyhydroxyalkanoate copolymer by *Alcaligenes denitrificans* A41

**DOI:** 10.1186/s13568-020-01093-5

**Published:** 2020-08-26

**Authors:** Taito Yajima, Mizuki Nagatomo, Aiko Wakabayashi, Michio Sato, Seiichi Taguchi, Michihisa Maeda

**Affiliations:** 1 Microbial Genetics Laboratory, Department of Agricultural Chemistry, School of Agriculture, Meiji University, 1-1-1 Higashimita, Tama-ku, Kawsaki, Kanagawa 214-8571 Japan; 2 Department of Chemistry for Life Sciences and Agriculture, Faculty of Life Sciences, Tokyo University of Agriculture, 1‐1‐1 Sakuragaoka, Setagaya, Tokyo 156–8502 Japan

**Keywords:** Bioremediation, Metabolic pathway, Polyester, Synthase, Two-step cultivation

## Abstract

A polyhydroxyalkanoate (PHA) copolymer, poly(3-hydroxybutyrate-*co*-3-hydroxyvalerate) [P(3HB-*co*-3HV)], was biosynthesized from biphenyl as the sole carbon source using *Alcaligenes* (currently *Achromobacter*) *denitrificans* A41. This strain is capable of degrading polychlorinated biphenyls (PCBs) and biphenyl. This proof-of-concept of the conversion of aromatic chemicals such as the environmental pollutant PCBs/biphenyl to eco-friendly products such as biodegradable polyester PHA was inspired by the uncovering of two genes encoding PHA synthases in the *A. denitrificans* A41 genome. When the carbon/nitrogen (C/N) ratio was set at 21, the cellular P(3HB-*co*-3HV) content in strain A41 reached its highest value of 10.1% of the cell dry weight (CDW). A two-step cultivation protocol improved the accumulation of P(3HB-*co*-3HV) by up to 26.2% of the CDW, consisting of 13.0 mol % 3HV when grown on minimum salt medium without nitrogen sources. The highest cellular content of P(3HB-*co*-3HV) (47.6% of the CDW) was obtained through the two-step cultivation of strain A41 on biphenyl as the sole carbon source. The purified copolymer had ultra-high molecular weight (weight-average molecular weight of 3.5 × 10^6^), as revealed through gel-permeation chromatography. Based on the genomic information related to both polymer synthesis and biphenyl degradation, we finally proposed a metabolic pathway for the production of P(3HB-*co*-3HV) associated with the degradation of biphenyl by strain A41. 
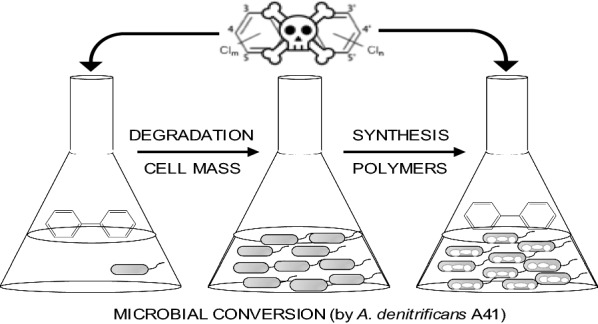

## Introduction

There are currently two biotechnological approaches for managing environmental problems: the bioremediation of target pollutants and the synthesis of eco-friendly products. As representative players of bioremediation approaches, *Achromobacter* and *Alcaligenes* spp. contain chromosomally encoded genes for the catabolism of phenol and catechol via the *meta*-cleavage pathway (Hinteregger and Streichsbier 2001; Collard et al. 1994). Some species have also acquired additional genes that extend their metabolic capabilities to include the degradation of polychlorinated biphenyls (PCBs) and other halogenated aromatic compounds (Springael et al. 1993; Springael et al. 1994). The degradation of these cytotoxic molecules typically proceeds through a series of dehalogenations and aromatic ring oxidations that convert the molecule into catechol, after which it enters into the *meta*-cleavage pathway and undergoes further metabolic conversion to acetate and pyruvate (Hughes and Bayly 1983).

A PCBs degrader*, Alcaligenes denitrificans* strain A41 is a Gram-negative, organic solvent tolerant, alkalitrophic, and denitrifying bacterium (Ohta et al. 1996; Maeda et al. 1998). The taxon of *Alcaligenes denitrificans* was reclassified as *Achromobacter denitrificans* (Coenye 2003). In our previous studies (Maeda et al. 1997; Tomizawa et al. 2015), a set of genes responsible for the metabolism of PCBs and biphenyl was found in the genome of *Alcaligenes denitrificans* strain A41.

While sequencing the whole genome of strain A41, we also found genes related to the synthesis of biodegradable polymers known as polyhydroxyalkanoates (PHAs) at different loci. This finding is in accordance with the fact that strain A41 is related to *Alcaligenes eutrophus* (currently *Ralstonia eutropha*), a known PHA producer (Schlegel et al. 1961; Raberg et al. 2018). Considering these findings, we aimed to establish a promising platform based on strain A41 for the conversion of PCBs and biphenyl to PHAs through a one-pot fermentation process by combining both degradation and synthetic pathways.

**Fig. 1:**
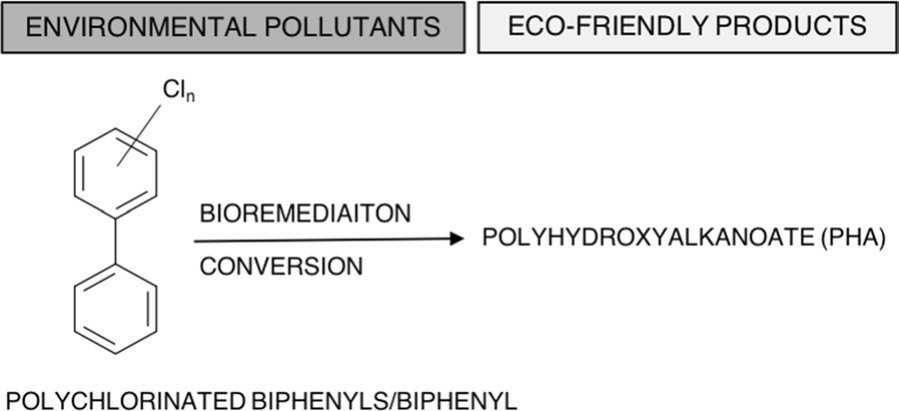
A conceptual scheme of the conversion of environmental pollutants, such as polychlorinated biphenyls/biphenyl, to eco-friendly products, such as polyhydroxyalkanoates (PHAs)

In the present study, this proof-of-concept (Fig. 1) conversion of xenobiotic compounds into value-added products such as PHAs was studied using nitrogen limitation conditions, as routinely performed for PHA production with *R. eutropha*. As a result, we achieved the biosynthesis of a PHA copolymer, poly(3-hydroxybutyarate-*co*-3-hydroxyvalerate) [P(3HB-*co*-3HV)] from biphenyl as the sole carbon source. The copolymer production was further improved by conducting a two-step fermentation with growth and polymer production phases. Moreover, we evaluated the molecular weight and monomeric composition of the biosynthesized polymer. Finally, we proposed a pathway for P(3HB-*co*-3HV) production from biphenyl based on our experimental results and on the available genome sequence information.

## Materials and methods

### Bacterial strains and media

*Alcaligenes denitrificans* A41 (deposited in WDCM567 as JCM9787) was cultured in Luria–Bertani (LB) medium (Sambrook et al. 2001) or C medium, which contained (per liter) 5 g of (NH_4_)_2_SO_4_, 2.93 g of KH_2_PO_4_, 5.87 g of K_2_HPO_4_, 0.3 g of MgSO_4_·7H_2_O, 2 g of NaCl, 0.03 g of CaCl_2_, 0.01 g of FeSO_4_· H_2_O, 0.6 mg of NiSO_4_·7H_2_O, and 2 mL of a trace element solution; the pH was adjusted to 7.0 using NaOH. The trace element solution contained (per liter) 4 mg of MoO_3_, 28 mg of ZnSO_4_·5H_2_O, 2 mg of CuSO_4_·5H_2_O, 4 mg of H_3_BO_3_, 4 mg of MnSO_4_·5H_2_O, and 4 mg of CoCl_2_·6H_2_O. Biphenyl was added to the C medium at a final concentration of 0.5% (not dissolved) or 0.3% as the sole carbon source, and cultures were incubated on a reciprocal shaker in 500-mL flasks containing 100 mL of the medium at 120 rpm and 30 °C. Antibiotics were adequately used as described previously (Sambrook et al. 2001).

For the one-step cultivation, the concentrations of nitrogen sources were altered to achieve a nutrient-deficiency condition that induced PHA production. For this, we used C medium containing 0.5% biphenyl as the sole carbon source and (NH_4_)_2_SO_4_ as the nitrogen source at concentrations changed by 1/10 (N-100), 1/20 (N-50), 1/40 (N-25) and 1/100 (N-10) of the original value (N-1000, C/N ratio = 2). These altered media had corresponding C/N ratios of 21, 41, 83, and 166, respectively.

In the two-step cultivation protocol, the first growth step, which was aimed at gaining cell mass, was performed on LB complex medium until the stationary growth phase was reached, and the second culture used C medium (without any nitrogen source) containing 0.5% biphenyl for PHA production. In between the two phases, cells were harvested using centrifugation and resuspended in the second medium.

In an ideal case, cells were grown using C medium containing 0.3% biphenyl as the first culture, then harvested after 36 h, and their initial dry cell weight was adjusted to 1.38 g/L for resuspension in C medium without nitrogen sources containing 0.3% biphenyl. This medium was then used for growth aiming PHA production.

Cells were harvested at several time points (from 6 to 108 h) by centrifugation at 4 °C and were then washed with distilled water and lyophilized.

### Polymer analysis using gas chromatography (GC) and gel permeation chromatography (GPC)

The cellular PHA content and polymer composition were determined by analyzing freeze-dried cells using GC. Approximately 15 mg of dry cells were subjected to methanolysis with a solution consisting of 1.7 mL methanol, 0.3-mL 98% sulfuric acid, and 2.0 mL chloroform at 100 °C for 140 min to convert the constituents to their methyl esters (Braunegg et al. 1978). The addition of 1 mL water to the reaction mixture induced phase separation. The lower chloroform layer was used for GC analysis on a B353B system (GL Sciences, Eindhoven, The Netherlands) equipped with an InertCap1 capillary column (30 m × 0.25 mm) and a flame-ionization detector.

The polymers accumulated in cells were extracted using chloroform for 48 h at room temperature and purified by reprecipitation with methanol. Their molecular mass was measured using a GPC 20A GPC system and 10A refractive index detector (Shimadzu, Kyoto, Japan) with Shodex K-806 M and K-802 columns. Chloroform was used as the eluent at a flow rate of 0.8 mL/min, and samples were applied at 1.0 mg/mL. Polystyrene standards with low polydispersity were used in the construction of a calibration curve.

### Biphenyl consumption analysis

Solid-state biphenyl was sterilized using ultraviolet radiation for 5 min on a clean benchtop. Strain A41 was cultured in a test tube with a screw cap containing 10 mL of 0.5% biphenyl (w/v). To evaluate the residual amount of biphenyl in triplicate at nine time points: 12 h, 24 h, 36 h, 48 h, 60 h, 72 h, 84 h, 96 h, and 108 h, the fraction of biphenyl was carried out as follows. To each culture sample, 10 mL of ethyl acetate (Wako Japan Co. Ltd., Osaka, Japan) was added and mixed well, and samples were then subjected to centrifugation (3300×*g* for 10 min.) using an MX-300 centrifuge (TOMY Co. Ltd., Tokyo, Japan). The supernatants were filtered with a polyvinylidene fluoride (PVDF) filter (13 mm diameter, pore size 0.22 µm, Merck Millipore, Burlington, USA). Samples were subjected to high-performance liquid chromatography (HPLC) for measuring the consumption of biphenyl as a solid compound during bacterial cultivation. The following set-up was used in our experiments: Gilson 305 and 306 pumps (Gilson, Lewis Center, USA); an Ascentis C18 15 cm × 4.6 mm, 5 µm column (SUPELCO, Apex Scientific, Kildare, Ireland), detection at 246 nm; and solvent composition consisting of H_2_O and acetonitrile (ratios of 3 and 7), and a flow rate of 1.0 mL/min. For biphenyl quantification, a benzene calibration curve was used as an internal reference.

### Transmission electron microscopy for the observation of polymer accumulation

Cells were harvested, washed twice in 0.1 M phosphate buffer (pH 7.0), and fixed in 2% (w/v) glutaraldehyde in the same solution. Subsequently, cells were suspended in 2% (w/v) OsO_4_ for 1 h, gradually dehydrated in ethanol [50%, 70%, 80%, 90%, and 99.5% (v/v); 15 min each], and embedded in Quetol651. Ultrathin sections (thickness 70 nm) were cut with a microtome using a Diatome diamond knife. The sections were collected using 400-mesh cupper grids coated with a carbon layer and observed in a Jeol‐2010 electron microscope (Jeol Ltd., Akishima, Japan).

### Genetic analysis

The functionality of the *phaC* gene from *A. denitrificans* A41 was evaluated using a gene disruption technique. The primers 5′-ATTCTAGACTGGACCCGGAATGCAAC-3′ and 5′-ATGGTACCCATCCCGCCTGTAACGTAAG-3′ were used to amplify the *phaC1* (ade) gene from the *A. denitrificans* A41 genome. The resulting amplicon was used as a probe for a subsequent colony hybridization. According to the available genome information for A41, the *phaC1B1R* locus can be efficiently cloned as a 5997-bp KpnI fragment. The bacterial chromosome was, therefore, digested with the KpnI endonuclease (Takara Bio, Shiga, Japan), a gel fragment containing approximately 6-kb DNA was cut out, and the extracted DNA fragments were cloned into the pUC19 vector (Takara Bio, Shiga, Japan). The DNA library obtained here was subjected to colony hybridization (Grunstein and Wallis 1979). Positive colonies selected from the library were used as candidates. Recombinant plasmids were extracted from these candidates and confirmed using Southern blot analysis (Southern 2006).

The DNA fragment containing tetracycline resistance gene were prepared by digestion of the pBR322 plasmid (Takara Bio Inc., Shiga, Japan) with EcoRI and StyI followed by blunting with T4 DNA polymerase. The resulting fragment was ligated into CpoI site in the *phaC1* gene of the KpnI fragment on vector pUC19 after digestion and blunting. The resulting plasmid was digested with KpnI, transformed into *A. denitrificans* A41 by electroporation (Micro Pulser, BIO-RAD, Hercules, USA), and candidates for the *phaC1* disruptant strain were selected on LB plates containing tetracycline. Colonies were selected from these plates, grown in LB medium containing tetracycline, and chromosomal DNA was extracted as described previously (Sambrook et al. 2001). The *phaC1*-disrupted strains were confirmed and selected using Southern blot analyses.

### Bioinformatic analysis of the A41 genes

The whole genome sequence of *A. denitrificans* A41 was determined by Takara Bio (Takara Bio Inc., Shiga, Japan). The *pha* genes and their neighboring sequences in the A41 genome were analyzed using the IMC GE software (available at https://www.insilico-biology.com). Nucleotide and amino acid (aa) sequences were retrieved from the National Center for Biotechnology Information (NCBI) database, and a phylogenetic tree was constructed based on the partial amino acid sequence (~ 596 aa) of PhaC using the maximum-likelihood method. Bootstrap values (1000 replicates) are shown for each branch.

### Genome sequence accession number

Draft genome sequences of *Achromobacter denitrificans* A41 were deposited into the DNA Data Bank of Japan with accession numbers BLWG01000001-BLWG01001041.

## Results

### Biosynthesis of P(3HB-*co*-3HV) by *A. denitrificans* A41 from biphenyl as a sole carbon source

**Fig. 2:**
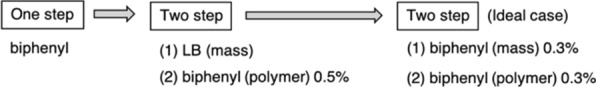
Growth conditions used for PHA production. First, a one-step cultivation method was used with biphenyl as the sole carbon source. Second, as a two-step procedure, initial cultivation was performed for gaining cell mass and the second step was conducted for polymer production. The highest polymer yield was obtained by two-step cultivation using biphenyl as the sole carbon source with controlled cell mass in the PHA production step. mass: culture for gaining cell mass, polymer: culture for PHA production

**Fig. 3:**
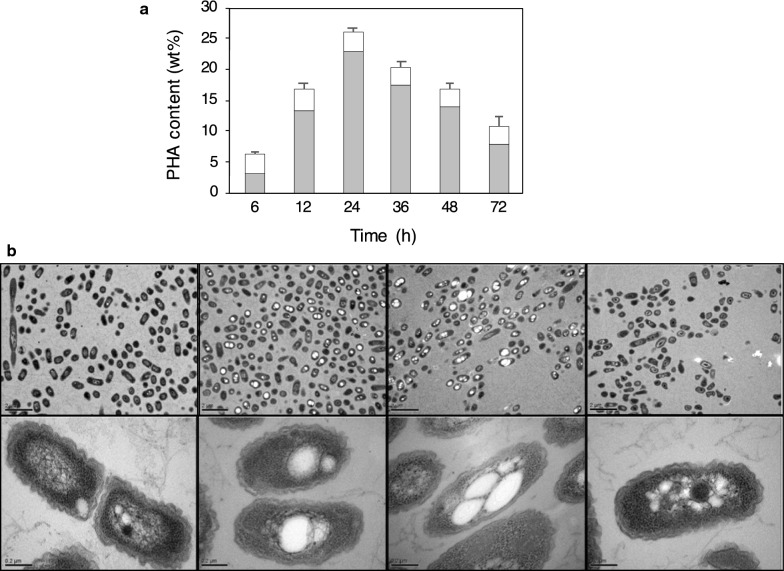
Polymer production throughout a 72-hour process (two-step cultivation). **a** Cellular contents sampled at the appointed times were analyzed. Gray and white boxes indicate 3HB and 3HV molar fractions, respectively. **b** Transmission electron micrographs showing the intracellular accumulation of polymer corresponding to the estimated polymer yields at the appointed times. Lower magnification (upper photos): 6 h, 12 h, 24 h, 72 h; higher magnification (lower photos): 6 h, 12 h, 24 h, 72 h

In order to evaluate the possibility of PHA production from biphenyl, we attempted to cultivate strain A41 on biphenyl as a carbon source. We succeeded in biosynthesizing P(3HB-*co*-3HV) from biphenyl using a C/N ratio of 21 (Table 1 and Fig. 2). Polymer accumulation was clearly observed using transmission electron microscopy (Fig. 3). This is the first report on the conversion of cytotoxic compound biphenyl to a PHA copolymer with a cellular copolymer content of 10.1% (Table 1).

**Table 1:** PHA productivity in the one-step cultivation process

C/N ratio	Cell dry weight (mg/L)	PHA content (%)	PHA composition (mol %)		PHA yield (mg/L)
			3HB	3HV	
166	5.5	8.6	30.6	69.4	4.2
83	13.2	5.9	16.5	83.5	6.4
41	31.1	4.0	6.0	94.0	10.1
21	47.9	10.1	54.4	45.6	47.3
2	60.9	4.3	5.2	94.8	26.4

The cellular content and molar fraction of the P(3HB-*co*-3HV) copolymer were measured using GC

### Improved production of P(3HB-*co*-3HV) from biphenyl through a two-step cultivation process

**Fig. 4:**
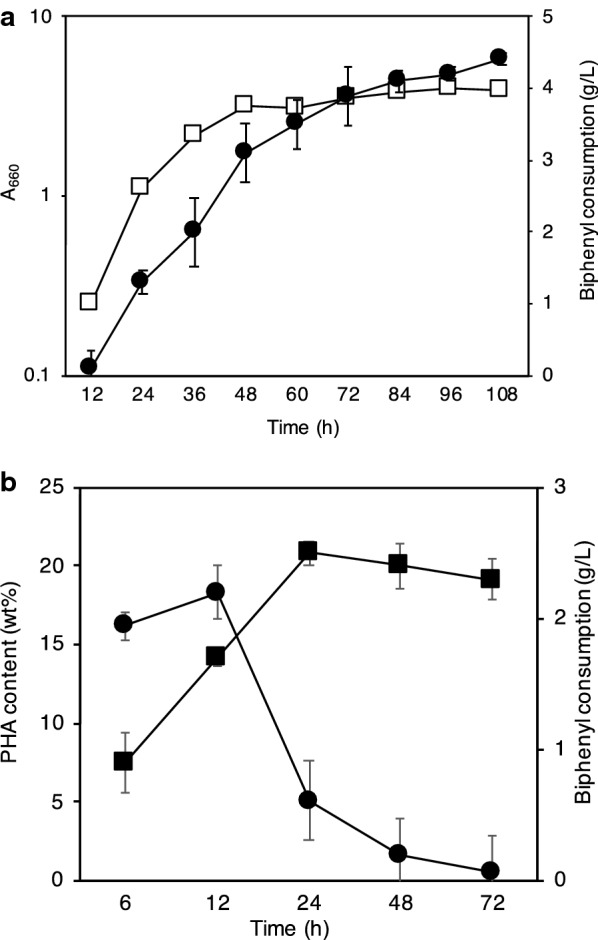
Biphenyl consumption and polymer production. **a** Relationship between cell growth and biphenyl consumption. Closed circles indicate biphenyl consumption. Open squares indicate cell growth. Cultivation time range is 120 h. **b** Relationship between PHA production and biphenyl consumption. Cultivation time range is 80 h. Closed circles indicate biphenyl consumption. Closed squares indicate PHA production. Bars indicate standard deviation in each graph

**Fig. 5:**
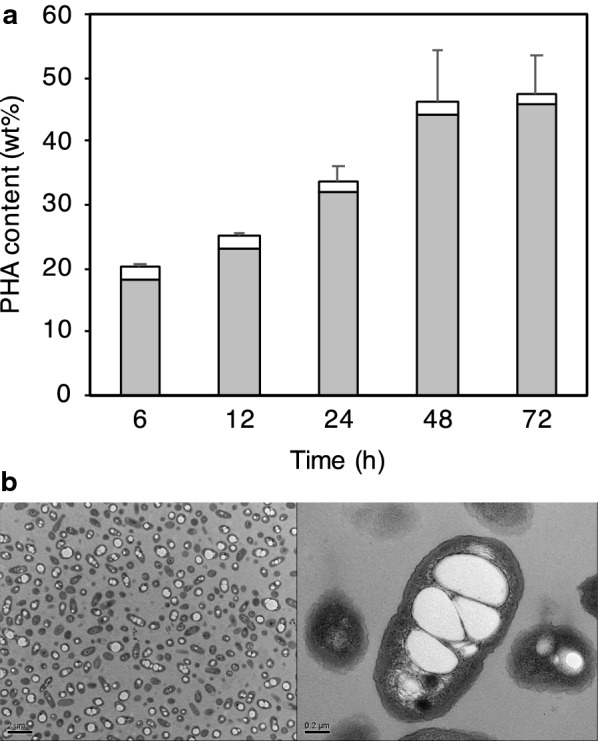
Time course of polymer production over 72 h (two-step cultivation process). **a** Cellular contents sampled at the appointed times were analyzed. Gray and white boxes indicate 3HB and 3HV molar fractions, respectively. **b** transmission electron micrographs showing the intracellular accumulation of polymer corresponding to the estimated polymer yields at 48 h. lower magnification (left photo); higher magnification (right photo)

In order to improve the cellular copolymer content, we used a two-step cultivation method with varying C/N ratios. This was performed to attain (a) cell growth for biomass formation, and (b) induction of PHA production under nitrogen limitation conditions by switching from LB rich medium to a poor medium. Expectedly, we obtained an increase in cellular polymer content (26.2%) when using nitrogen limitation (Table 2 and Fig. 3). The accumulation of polymer was clearly observed using transmission electron microscopy (Fig. 3). Figure 4a shows the time course of biphenyl consumption associated with cell growth by biphenyl assimilation. The biphenyl consumption rates were 0.080 g/h (from 12 h to 48 h) and 0.014 g/h (from 72 h to 108 h), respectively. However, biphenyl was also used for production of P(3HB-*co*-3HV). Its consumption rate was 0.087 g/h (from 6 h to 24 h) (Fig. 4b). The highest cellular content (47.6%) of polymer was obtained by two-step cultivation of stain A41 on biphenyl as the sole carbon source (Fig. 5).

**Table 2:** PHA production in the two-step cultivation process (24 h)

C/N ratio	Cell dry weight (mg/L)	PHA content (%)	PHA composition (mol %)		PHA yield (mg/L)
			3HB	3HV	
–	1.15	26.2	87.0	13.0	298
166	1.21	21.3	88.5	11.5	266
21	1.71	4.1	33.6	66.4	70.0
2	1.23	3.1	12.6	87.4	38.7

The cellular content and molar fraction of the P(3HB-*co*-3HV) copolymer were measured using GC

### Functional analysis of a PHA synthase, PhaC1, encoded in the *A. denitrificans* A41 genome

**Fig. 6:**
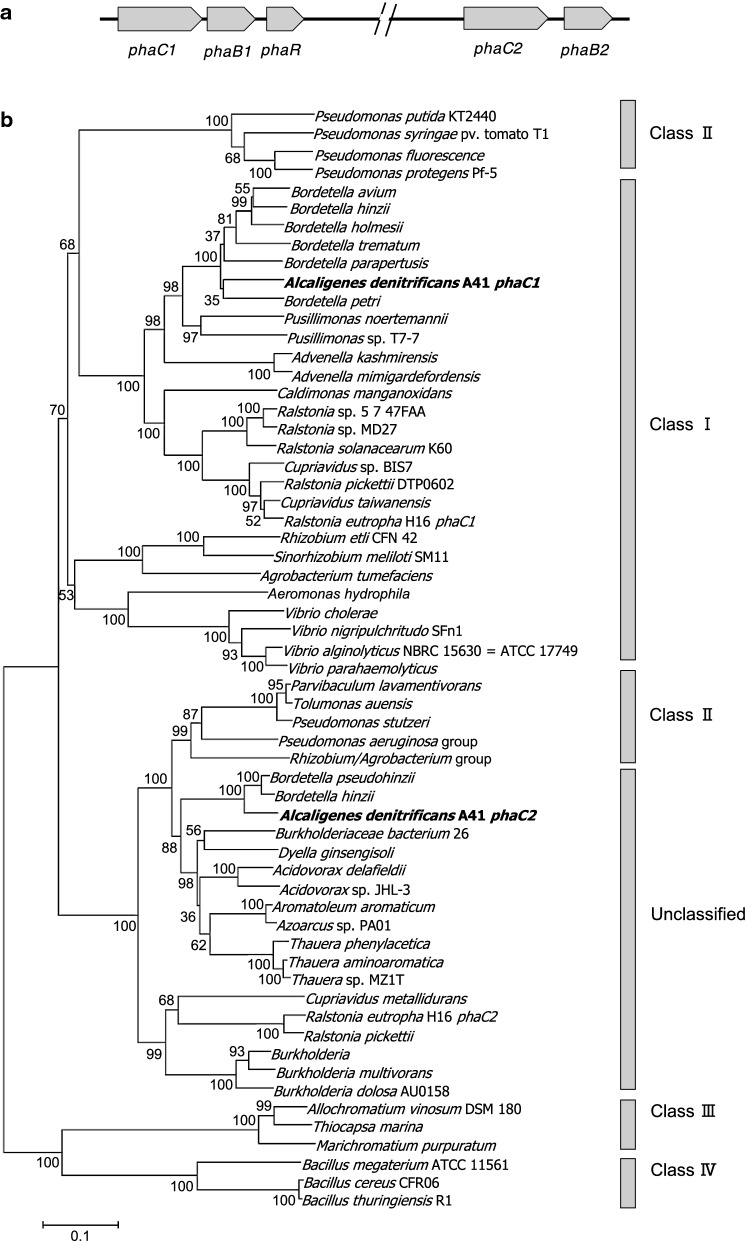
Organization of PHA synthesis-related genes and phylogenetic tree of PHA synthases. **a** Gene organization of PHA synthesis-related enzymes in the *A. denitrificans* A41 genome. Putative PHA synthesis-related genes are termed as *phaC1* and *phaC2* (encoding PHA synthases), *phaB1* and *phaB2* (encoding NADPH-dependent reductases), and *phaR* (encoding a regulatory protein). **b** Using Mega 6.0 software, a phylogenetic tree was constructed for classifying a putative PhaC1 of strain A41 based on the amino acid sequences of PHA synthases

Sequences for two possible PHA synthases were found in different loci of the *A. denitrificans* A41 genome. Figure 6a shows the genomic organization of genes related to PHA synthesis. Figure 6b shows the phylogenetic relationship among PHA synthases based on the multiple alignment of amino acid sequences. From this phylogenetic tree, PhaC1 can be categorized as a class I PHA synthase, while PhaC2 remains unclassified. The putative functionality of PhaC1 was evaluated by disrupting the corresponding gene. A negative result for polymer production demonstrated the functionality of PhaC1 (data not shown).

## Discussion

To date, many studies on the utilization of renewable carbon sources have been reported for the production of value-added products such as PHAs (Matsumoto and Taguchi 2013; Kourmentza et al. 2017). The use of xenobiotic compounds as carbon sources for future bioprocesses would be preferable as a solution for environmental problems. The following aromatic compounds have been reported as environmental pollutants: polycyclic aromatic hydrocarbon (Sangkharaket al. 2020) and lignin (Salvachüa et al. 2020). In this study, PCBs/biphenyl were chosen as promising targets and PHA production was achieved from biphenyl as the sole carbon source using *A. denitrificans* A41, which is capable of degrading PCBs/biphenyl.

In this study, PHA production by strain A41 was first demonstrated under varying C/N ratio conditions using a one-step cultivation process. The amount of cell mass obtained through this process was low and PHA production was not expressive. Thus, a two-step cultivation process was used in order to improve PHA productivity. In this case, it was important to address the consumption of biphenyl considering two fermentative events: cell growth and PHA production.

It is well known that the precise quantification of biphenyl is not easy due to the high volatility of this compound. As shown in Fig. 4a, biphenyl was constantly consumed for cell growth until 48 h of culture, which corresponded to the beginning of the stationary phase. The consumption rates remarkably decreased after 72 h. Approximately 96% of the added biphenyl was consumed after 108 h. This kinetic transition of biphenyl consumption (from 48 h to 72 h) suggests that cell growth was probably decreased because of the generation of a cytotoxic benzoic acid as an intermediate metabolite.

The next aspect is the feasibility of the use of biphenyl for PHA production. The cellular PHA content was 18.3% after 12 h of cultivation under nitrogen limitation conditions (Fig. 4b). Furthermore, the highest cellular content (47.6%) was obtained by two-step cultivation with biphenyl as the sole carbon source (Fig. 5). By controlling culture conditions, the conversion from biphenyl to P(3HB-*co*-3HV) was achieved by the strain A41. This successful result may indicate that initial cell growth should first be achieved before PHA production, preferably reducing the cytotoxicity of biphenyl. Regarding the molecular weight of the polymer product, much higher values were obtained than those obtained in the other cases such as the conversion of aromatic hydrocarbon styrene to PHA (Ward et al. 2005; Tan et al. 2015; Arshad et al. 2017). It is of interest to address the reason behind this in our microbial conversion system.

The biosynthesis of copolymer P(3HB-*co*-3HV) can also be supported by the amino acid sequence deduced from genes encoding PHA synthase PhaC1, which are possibly categorized into the class I PHA synthase family (Fig. 6b). Class I PHA synthases could exhibit substrate specificity toward short-chain-length monomers, 3HB and 3HV (Rehm 2003). PhaC1 could at least be involved in polymer biosynthesis, as demonstrated by our gene disruption experiment. The precursor CoA forms 3HB-CoA and 3HV-CoA are generated via two condensation reactions: one with two molecules of acetyl-CoA and the other with one molecule of acetyl-CoA and one molecule of propionyl-CoA, respectively (Anderson and Dawes 1990). A possible regulator protein (PhaR), encoded downstream to the *phaC1* gene, could be a promising target for future PHA biosynthesis strategies.

**Fig. 7:**
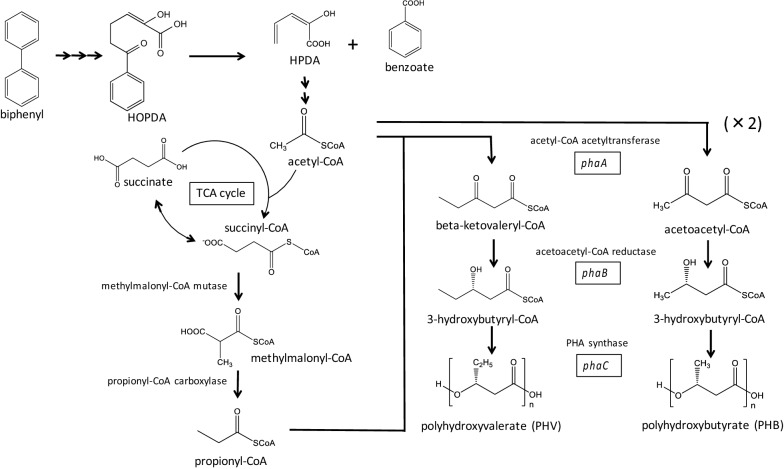
Proposed pathway for PHA production associated with biphenyl degradation. The biodegradation pathway for biphenyl (shown to the left) has already been described (Brenner et al. 1994). HOPDA, 2-hydroxy-6-oxo-phenylhexa-2,4-dienoate, is degraded to HPDA, 2-hydroxypenta-2,4-dienoate, and benzoate. HPDA is metabolized to pyruvate and acetyl-CoA, and the pyruvate is further converted to acetyl-CoA through common cellular functions such as that by pyruvate dehydrogenase. Acetyl-CoAs generated here would be used to produce polyhydroxybutyrate (PHB) via β-ketothiolase (encoded by *phaA*), NADPH-dependent reductase (encoded by *phaB*), and PHA synthase (encoded by *phaC1*). As for the generation of 3HV-CoA, it can be speculated that a possible sequential conversion from succinyl-CoA to methylmalonyl-CoA, and consequently to propionyl-CoA, would be carried out by the intracellular metabolism. Finally, 3HV-CoA would be generated by the condensation of propionyl-CoA with acetyl-CoA. Accordingly, copolymer P(3HB-*co*-3HV) can be biosynthesized by the copolymerization of two molecules, 3HB-CoA and 3HV-CoA

Considering these findings, we propose a hypothetic pathway for PHA production associated with a previously described biphenyl degradation pathway (Brenner et al. 1994), as illustrated in Fig. 7. Biphenyl is converted to 2-hydroxy-6-oxo-phenylhexa-2,4-dienoic acid (HOPDA) by BphA, BphB, and BphC; benzoic acid and 2-hydroxypenta-2,4-dienoic acid (HPDA) by BphD. Among these compounds, HPDA is metabolized to pyruvate and acetyl-CoA, and this pyruvate is further converted to acetyl-CoA by intracellular enzymes. These acetyl-CoA molecules are used to produce polyhydroxybutyrate (PHB) by β-ketothiolase (encoded by *phaA*), NADPH-dependent reductase (encoded by *phaB*) and PHA synthase (encoded by *phaC1*). Considering the generation of 3HV-CoA, it can be speculated that a possible sequential conversion from succinyl-CoA to methylmalonyl-CoA, and consequently to propionyl-CoA, would be carried out through intracellular metabolism. Finally, 3HV-CoA would be generated by condensation of propionyl-CoA with acetyl-CoA. Accordingly, copolymer P(3HB-*co*-3HV) could be biosynthesized by the copolymerization of two molecules, 3HB-CoA and 3HV-CoA.

In conclusion, the native strain A41 provided here the prototype of a microbial platform for the production of copolymer P(3HB-*co*-3HV) by supplying a cytotoxic biphenyl. In the near future, a recombinant technology (Zhou et al. 2020) will be applied for the strain A41 platform, in order to improve PHA productivity and alter properties related to molecular weight and monomeric composition.

## Data Availability

All the relevant data used to support the findings of this study are included within the article.
